# Characterization of a Full-Length Endogenous Beta-Retrovirus, EqERV-Beta1, in the Genome of the Horse (*Equus caballus*)

**DOI:** 10.3390/v3060620

**Published:** 2011-06-01

**Authors:** Antoinette C. van der Kuyl

**Affiliations:** Laboratory of Experimental Virology, Department of Medical Microbiology, Centre for Infection and Immunity Amsterdam (CINIMA), Academic Medical Centre of the University of Amsterdam, Meibergdreef 15, 1105 AZ Amsterdam, The Netherlands; E-Mail: a.c.vanderkuyl@amc.uva.nl; Tel.: +31-20-5666778; Fax: +31-20-5669064

**Keywords:** *Equus caballus*, horse, endogenous virus, full-length, beta-retrovirus

## Abstract

Information on endogenous retroviruses fixed in the horse (*Equus caballus*) genome is scarce. The recent availability of a draft sequence of the horse genome enables the detection of such integrated viruses by similarity search. Using translated nucleotide fragments from gamma-, beta-, and delta-retroviral genera for initial searches, a full-length beta-retrovirus genome was retrieved from a horse chromosome 5 contig. The provirus, tentatively named EqERV-beta1 (for the first equine endogenous beta-retrovirus), was 10434 nucleotide (nt) in length with the usual retroviral genome structure of 5′LTR-gag-pro-pol-env-3′LTR. The LTRs were 1361 nt long, and differed approximately 1% from each other, suggestive of a relatively recent integration. Coding sequences for gag, pro and pol were present in three different reading-frames, as common for beta-retroviruses, and the reading frames were completely open, except that the env gene was interrupted by a single stopcodon. No reading frame was apparent downstream of the env gene, suggesting that EqERV-beta1 does not encode a superantigen like mouse mammary tumor virus (MMTV). A second proviral genome of EqERV-beta1, with no stopcodon in env, is additionally integrated on chromosome 5 downstream of the first virus. Single EqERV-beta1 LTRs were abundantly present on all chromosomes except chromosome 24. Phylogenetically, EqERV-beta1 most closely resembles an unclassified retroviral sequence from cattle (*Bos taurus*), and the murine beta-retrovirus MMTV.

## 1. Introduction

Vertebrate genomes generally contain large numbers of elements that were acquired by the host species over time, including variable numbers of integrated viral genomes. Genomic counterparts of borna-, ebola-, parvo- and filovirus genomes have been found in different species [1–3] as well as integrated/Mendelian transmitted herpesvirus (HHV-6) genomes in humans [4]. The first discovered and best described of the integrated viral genomes are endogenous retrovirus proviral sequences (reviewed in [5]). Most classes of retroviruses, comprising of simple (alpha-, beta-, gamma-, and epsilon-retroviruses), and more complex (spuma-, and lenti- retroviruses), have been identified in vertebrate genomes [6–9]. By now, many extant species have been analyzed for their endogenous retrovirus content, and even the extinct woolly mammoth has been shown to contain endogenous proviral fragments in its genome [10]. Surprisingly, data for the domestic horse (*Equus caballus*) are scarce. A few short pol-gene fragments with similarity to foamy viruses are the only endogenous retrovirus sequences from horses published today [11].

Recently, a high-quality draft sequence of the horse genome has been published [12], and is available for Basic Local Alignment Search Tool (BLAST) searches through the Horse Genome Resources website of the NCBI and for BLAT (The BLAST-like Alignment Tool) searches through the Horse (*Equus caballus*) Genome Browser Gateway of the Genome Bioinformatics Group of UC Santa Cruz [13].

## 2. Experimental Section

Horse genomic sequences were available from the NCBI website [14] and from [13]. BLAST [15] and BLAT (The BLAST-like Alignment Tool [13] searches were performed with translated protein sequences from gamma- and beta-retroviruses retrieved from the NCBI nucleotide database [16]. Retrieved sequences were analyzed with BioEdit Sequence Alignment Editor version 7.0.9 [17]. Phylogenetic analysis was performed with the Neighbor-joining option in MEGA [18].

## 3. Results

### 3.1. Detection of Endogenous Retrovirus pol Fragments in the Horse Genome

Searching the translated horse genome with translated polymerase gene fragments of exogenous gamma- and beta- retroviruses encompassing the highly conserved YXDD (where X = M, V, or I) motif (TBLASTN option) revealed high numbers (>200) of homologous sequences distributed over all horse chromosomes, including the X chromosome. However, the Y chromosome could not be queried as the draft sequence was generated from a mare. Highest similarities were found for murine leukemia virus (MuLV, acc. no. DQ366149, query = 1731 amino acid gag-pro-pol, best hit 43% identities/ 1192 amino acid fragment on chromosome 2), baboon endogenous virus (BaEV, acc. no. D10032, query = 1726 amino acid gag-pro-pol, best hit 47% identities/ 772 amino acid fragment on chromosome 20), mouse mammary tumor virus (MMTV, acc. no. AF033807, query = 895 amino acid pol, best hit 59% identities/ 850 amino acid fragment on chromosome 5) and simian Mason-Pfizer type D virus (MPMV, acc. no. M12349, query = 874 amino acid pol, best hit 52% identities/850 amino acid fragment on chromosome 5). The MMTV and MPMV highest scoring BLAST hit was identical in location. Searching the horse genome with translated gag or env sequences always generated lower % identities and e-values, as would be expected for these less conserved proteins.

### 3.2. A Complete Beta-Retrovirus Genome Is Integrated on Horse Chromosome 5

Next, the chromosome locations with the highest scoring BLAST hits for pol were analyzed for the presence of flanking long terminal repeat (LTR) regions. Corresponding regions were downloaded as fasta-files from the database and a sequence of up to 3000 nucleotides in front of the pol fragment was compared with the complete segment by using the “align two sequences” option (bl2seq) on the NCBI BLAST website. In addition, the downloaded segments were analyzed for the presence of a primer binding site (PBS), which is essential for viral replication and is located directly downstream of the 5′LTR, using BioEdit [17] and PBS sequences for PBS(Trp), PBS(Pro), PBS(Lys1,2), PBS(Lys3), and PBS(Phe) (for a review on PBS sequences, see [19]).

**Figure 1.**
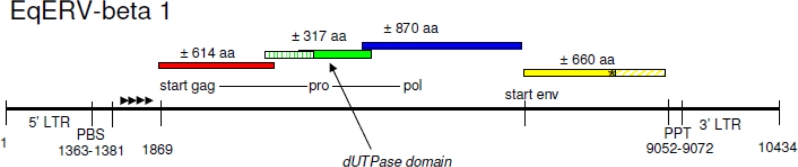
Schematic representation of the genome organization of the horse endogenous retrovirus EqERV-beta1 on chromosome 5. The figure is not drawn to scale, but important features and reading frames are indicated. A stopcodon in env is marked with an asterisk. The length of the provirus is 10434 nt. Four direct repeats located between the PBS and the startcodon of gag-pro-pol are indicated by arrowheads. LTR = long terminal repeat; PBS = primer binding site; PPT = polypurine tract.

Horse chromosomal contigs containing a gamma-retroviral pol fragment did not contain a linked putative 3′LTR or a PBS sequence upstream of pol (not shown). Also, gag and env genes were difficult to identify, and are possibly either highly fragmented or deleted. BLAST scores to beta-retroviral genes, including the env gene, were generally higher. In addition, a putative LTR sequence was detected on chromosome 5 followed by an intact PBS(Lys3) sequence. Similarity to the MMTV env gene of this putative provirus was 155/396 amino acid identity (40%) of a 591 amino acid query. A next BLAST search using the putative 5′LTR sequence combined with 5′ TGGCGCCCGAACAGGGAC 3′(= PBS(Lys3)), revealed only a single location in the horse genome with an LTR + PBS (5′LTR) and an LTR without PBS (3′LTR), separated by approximately 8–9,000 nucleotides, the size of a retroviral genome. A segment from chromosome 5 (GenBank acc. no. NW_001867417.1) containing this putative provirus, was retrieved and analyzed in more detail. Indeed, a complete provirus of 10434 nucleotides (nt) with homology to the beta-retrovirus genus was present on this segment in the reverse orientation from nucleotide positions 2009202 till 1998769. The expected proviral structure (5′LTR-gag-pro-pol-env-3′LTR) was completely intact, and was named Equus Endogenous RetroVirus EqERV-beta1, for the first equine endogenous beta-retrovirus to be described. A schematic representation of EqERV-beta1 is shown in Figure 1.

### 3.3. Elements Related to EqERV-Beta1 in the Horse Genome

Searching the horse genome database with sequences of EqERV-beta1 as probe revealed one single complete provirus of this genus in the assembly of February 2011. However, around 20,000 nucleotides downstream of the complete provirus on chromosome 5, a second complete proviral integration with different sequences flanking the integration sites is present, also in the reverse orientation. Unfortunately, this structure cannot be extensively characterized yet, as difficulties in assembly and stretches of ambiguous nucleotides trouble the draft horse genome sequence in this location. It is useful, however, to compare the viral features of the two integrations.

**Figure 2.**
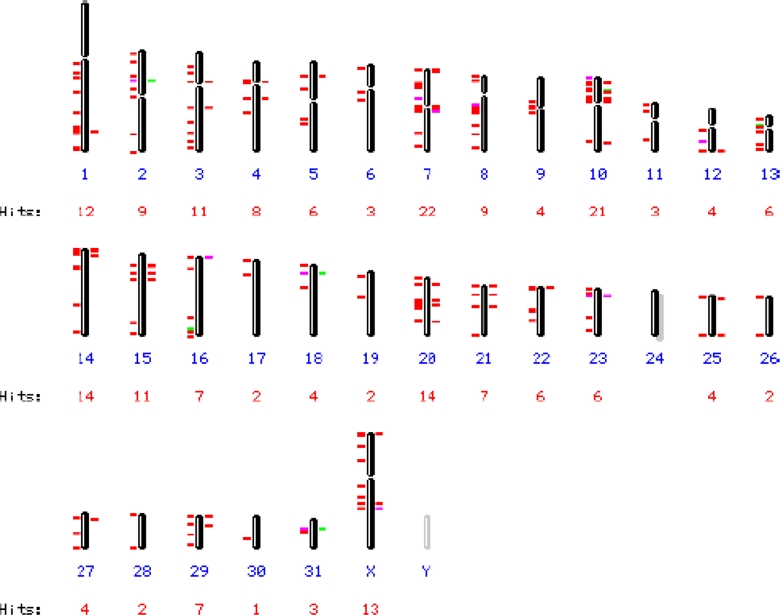
Distribution of EqERV-beta1 long terminal repeat (LTR) sequences over the horse chromosomes. NCBI Map Viewer output of a BLAST search of the horse (*Equus caballus*) genome (2N = 64) with the EqERV-beta1 LTR as query sequence. Chromosome numbers (blue) and hits per chromosome are indicated. Only 15 of 227 LTRs, distributed over 10 chromosomes, did not correspond to a full-length LTR or showed a lower sequence homology (generally 85–89%, indicated with a green or pink line), while most integrations were >95% homologous to the query sequence (indicated with a red line).

EqERV-beta1 5′and 3′ LTRs including short sequences downstream and upstream of the LTRs are present on chromosomes 7 and 20, but the coding regions of these integrations are missing. Blasting only the LTR region revealed 227 integrations with high homology (>80%, mostly >95%) assigned to 31/32 chromosomes, including the X chromosome (Figure 2), the Y chromosome could not be investigated. Only chromosome 24 did not contain any EqERV-beta1 LTR sequences. The largest number of hits (22) was found on chromosome 10, with chromosome 5 harboring six LTRs. Four LTRs, on chromosomes 5 (2×), 7 and 20, were followed by a PBS(Lys3) sequence.

### 3.4. The LTR of the Horse Endogenous Retrovirus EqERV-Beta1

LTR sequences of the provirus started with the sequence TG and ended with CA as usual in retroviral integrations [20]. The LTRs were 1361 nt in length and differed at 14 nt positions, with two additional 1 nt deletions (≈ 1% variation). Assuming a nucleotide substitution rate of 10^−8^ substitution/base pair/generation, as calculated for the human genome [21] and a generation time of three years for the horse, this corresponds to a relatively recent integration event dating approximately 300,000 years ago, as retroviral LTRs are identical at the moment of integration. Beta-retrovirus LTRs are the longest known amongst retroviruses, with a length exceeding 1000 nt. Conserved elements (see [22]) like a TATA box (nt 1217-1224: TATATAAA), an AATAAA motif (nt 1239-1244: AGTAAA) and a C/T rich stretch can be recognized in the LTR of EqERV-beta1. The 5′ LTR was followed by a completely conserved PBS(Lys3), and reading frames for gag, pro, pol and env. The 3′ LTR was preceded by a 21 nt long polypurine tract (PPT, 5′A_5_GTA_6_G_5_AGA 3′) involved in plus-strand DNA synthesis, that was also found to be 100% conserved adjacent to 3′LTR’s integrated on chromosomes 7, 20 and 21 (BLAST result not shown).

### 3.5. Analysis of the Reading Frames of the Horse Endogenous Retrovirus EqERV-Beta1

A startcodon for gag-pro-pol translation is present at nucleotides 1869–1871, almost 500 nt downstream of the PBS. The sequence between the PBS and the startcodon of gag contains four repeated domains which each consist of an almost identical sequence followed by a 6–11 TAA repeat motif. This fragment is identical to unpublished equine DNA fragments labeled equine microsatellite DNA (e.g., Genbank acc. no. FN419635), but is also part of other, related proviral structures on *E. caballus* chromosomes 7 and 20 which contain an identical 5′ LTR + PBS(Lys3) upstream of a same repeat segment (BLAST result not shown, sequence identity 96–98%, 1–2% gaps). Also, a putative second EqERV-beta1 provirus on chromosome 5 with 99.6% homology also possesses this structure downstream of its 5′ LTR. This suggests that the repeat fragment is not an artifact of this specific integration, but has indeed been part of the replicating virus genome. In MMTV, the region between PBS(Lys3) and the gag startcodon is much shorter (around 160 nt in GenBank acc. no. AF228552 that contains full-length LTRs), and very T-rich, but contains no simple repeats.

Gag, pro and pol were encoded in three different reading frames, which is typical for beta-retroviruses. The putative reading frames for these three proteins are completely open. The alleged sizes for gag, pro and pol are respectively 614, 317 and 870 amino acids. Because the gag-pro-pol gene is translated by ribosomal frameshifting, exact N- and C-terminal ends of the proteins are difficult to assign, but the deduced lengths are similar to other beta-retroviruses. The 5′ end of the protease ORF contains a dUTPase domain, which is common in many retrovirus families, including the beta-retroviruses [23]. All five amino acid motifs normally seen in dUTPases are completely conserved in this provirus [24].

An env reading frame is present at the 3′ end of the genome, which can encode a protein of 692, 662 or 660 amino acids, depending upon which startcodon is used. The first two putative startcodons of env are not found in an mRNA with similarity to EqERV-beta1 (GenBank acc. no. XM_001914834). This suggests that translation of env starts at the third codon (MRRLSLR). An envelope gp41-like subunit domain was found by similarity search starting at amino acid 459 from the third start codon [25].

Two in-frame startcodons are present, one before the stopcodon of pol, and one after this stopcodon. The env ORF is interrupted by a single stopcodon, leading to translation termination at amino acid position 442 (TGG (Trp) → TGA at position 8359 of the provirus). In the putative second provirus on chromosome 5, the env reading frame is not interrupted by such a stopcodon and is completely open. No significant reading frame preceded by a startcodon is found downstream of the env gene, suggesting that EqERV-beta1 does not encode a MMTV-like superantigen. At present, no beta-retrovirus other than MMTV is known to encode such a superantigen.

### 3.6. Phylogenetic Analysis of EqERV-Beta1

**Figure 3.**
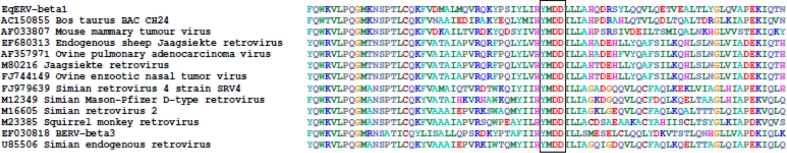
Alignment of translated pol sequences of EqERV-beta1 and reference beta- and delta-retroviruses. A pol fragment, corresponding to amino acid positions 147-226 in EqERV-beta1, surrounding the conserved YMDD motif (boxed) is shown. Accession number AC150855 (BAC CH240-472P12 from *Bos taurus*) harbours a further unidentified endogenous retrovirus pol sequence (nt 6344-3579, minus strand) with large homology to EqERV-beta1 (as detected by a BLAST search of the entire nucleotide collection).

**Figure 4.**
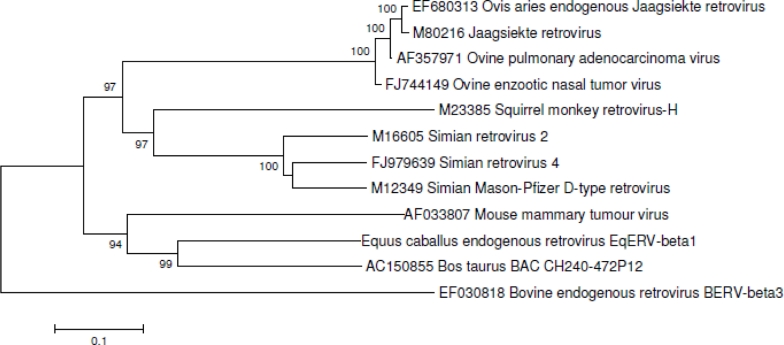
Phylogenetic analysis of the translated pol gene of EqERV-beta1 and reference translated pol sequences from beta- and delta-retroviruses was performed using the Neighbor-Joining option in MEGA4.0 with a Poisson distribution of amino acid substitutions and equal rates among sites. Five hundred bootstrap replicates were analyzed. Bootstrap values >90 are shown. Sequences had been aligned using the Clustal W option as implemented in BioEdit [17], and alignments were adjusted manually. GenBank accession numbers are indicated.

Phylogenetic analysis of the translated pol gene of EqERV-beta1 was performed using the Neighbor-Joining option in MEGA4.0 and reference sequences from beta- and delta-retroviruses. An aligned fragment of the pol gene surrounding the conserved YMDD motif is shown in Figure 3. EqERV-beta1 pol most closely resembles a not yet described retroviral integration from the cow (Accession number AC150855, BAC CH240-472P12 from *Bos taurus*) that was retrieved after BLASTing the NCBI nucleotide collection with the EqRV-beta1 pol sequence, and both strongly cluster together with MMTV (Figure 4). Beta-retroviruses from sheep and goats are only distantly related to EqERV-beta1, as is another retrovirus from cattle, BERV-beta3.

## 4. Discussion and Conclusions

Searching the draft version of the horse genome for endogenous retrovirus sequences revealed that gamma- and beta-retroviral elements are abundant. However, gamma-retroviral integrations were fragmented, and no intact provirus was found, suggesting that infection of the horse ancestor with gamma-retroviruses occurred a long time ago. A full-length beta-retrovirus was detected on horse chromosome 5. The provirus that was named EqERV-beta1 is the first endogenous equine beta-retrovirus. It is the result of a relatively recent integration, and conserves almost complete coding capacity; only a single stop codon is found in the env gene. As this stop codon is not seen in mRNA isolated from horse tissue, it might be a sequencing artifact. No other full-length EqERV-beta1 proviruses are found on other chromosomes. The overwhelming presence of single LTRs with very high homology to EqERV-beta1 suggests that an ancestor of the modern horse experienced massive integration of an infecting beta-retrovirus at a relatively short period in evolution less than possibly 0.5 million years ago, but was able to eliminate most coding regions from its DNA. Although horse-breeds from around the world are closely related [12], it could be that haplotypes differ with respect to EqERV-beta1 integrations, and additional elements could be present in breeds other than Thoroughbreds from which the draft genome was generated. It might also be interesting to look for EqERV-beta1 homologues in the donkey (*Equus asinus*), a related species that did not share geography with horses in the recent past (it does so now after domestication, however).

EqERV-beta1 is most similar to an unclassified endogenous retrovirus from the bovine genome, and to MMTV, a murine retrovirus. Horses and cattle are large grazing animals that shared habitat and geography in the recent past, so it is not remarkable that both species were infected by a similar virus strain. It is, however, striking that these ungulates were probably infected with a murine virus, as the phylogenetic analysis suggests that MMTV is ancestral to the ungulate viruses. Most likely, many novel retrovirus strains that once represented infectious viruses will be discovered in the near future as more and more genomes of different species are sequenced.
